# Detection of spatial, temporal and spatiotemporal distribution of diarrhea incidence among under-five children in Central Gondar zone, Northwest Ethiopia: a time-series study (2019–2022)

**DOI:** 10.1186/s12887-024-04900-1

**Published:** 2024-07-05

**Authors:** Gelila Yitageasu, Hailemariam Feleke, Zewudu Andualem, Lidetu Demoze, Kidist Asrat, Zemichael Gizaw

**Affiliations:** Department of Environmental and Occupational Health and Safety, Institute of Public Health, College of Medicine and Health Sciences, University of Gondar, Gondar, Ethiopia

**Keywords:** Diarrhea incidence, Under-5 children, Spatial, Temporal, Spatiotemporal, Distribution

## Abstract

Under-five children’s diarrhea is a significant public health threat and the World Health Organization (WHO) reported it as the second leading cause of children’s death worldwide. In this study area, little is known about the spatiotemporal distribution of under-5 diarrhea incidence. This study was therefore, conducted among all districts in the Central Gondar zone to assess the spatial, temporal, and spatiotemporal variation in diarrhea incidence among under-five children in the Central Gondar zone. The data for children under 5 years of age with diarrhea was obtained from Central Gondar Zone Health Department diarrhea reports from January 2019 to December 2022. All districts were included and geo-coded. The spatial data were created in ArcGIS 10.8.1. Global and local spatial autocorrelation were used to detect hot spots and cold spots. The Poisson model was generated by applying the Kulldorff method in SaTScan™9.6 to analyse the the purely temporal, spatial, and space-time clusters. The study revealed spatial variation of under-5 diarrhea where Gondar City, Gondar Zuria, East Dembia, and Lay Armacho districts were the high-rate spatial clusters during the study period. A year search window for temporal scan statistic identified 01 January 2020-30 December 2021 as risk periods across all districts. Spatiotemporal scan statistics detected high-rate clusters at Gondar City, Gondar Zuria, East Dembia, Lay Armacho, and Alefa between 2019 and 2022. In conclusion, there has been a spatial, temporal, and spatiotemporal variability of under-5 children’s diarrhea in the Central Gondar Zone. Interventional and preventive strategies should be developed and given priority to the areas that has been detected as a hotspot in this study to reduce the mortality and morbidity of under 5 children.

## Introduction

Globally diarrhea is responsible for the major causeof death in children under-5 years aged. It kills more children than AIDS, malaria, and measles combined [1]. Globally it causes 8% of deaths among under-5 [2]. Since 2022, more than 1,300 young children are dying each day, or approximately 484,000 children a year, despite the availability of a simple treatment solution [3]. Even if there has been a significant decline in childhood mortality rates from diarrhea, it still poses a major public health concern, especially in developing countries [4–6]. Among children under-5years of age, the number of deaths caused by diarrhea is highest in South Asia and sub-Saharan Africa [3]. Ethiopia was ranked fifth worldwide in terms of total child deaths and approximately 73,700 children die each year due to diarrhea which accounts for 20% of all child deaths [7, 8].

Diarrhea is highly prevalent in Ethiopia and is the second leading cause of death [9, 10]. The prevalence decreased from 24% in 2000 to 18% in 2005 [11], 13% in 2011 [12], and 12% in 2016 [13]. According to the recent EDHS, the incidence of childhood diarrhea was 11.87% which is high in the SNNP, Amhara, Addis Ababa, and Oromia regions [14]. The prevalence varied from 8.5 to 30.5% in different parts of the country. Moreover, the yearly number of childhood deaths due to diarrheal illness was estimated to be 95,000 [15]. In 2021 the mortality rate was 46.8 per 1000 live births [16].

The incidence of diarrhea is highly heterogeneous both within and between countries, exhibiting substantial spatial and temporal variability [17]. For example, stud ies conducted in Ethiopia Azage et al. [18]. and Bench Maji Zone [19] found a spatial and temporal variation of under-5 diarrhea in different parts of Ethiopia,. These variations are largely driven by meteorological factors [20], socioeconomic status and WASH infrastructure [21], poor hygiene practices and unsafe human waste disposal [22], and access to and quality of health services [23]. The interaction of these factors can lead to different pathways for diarrhea transmission, which creates a setting-specific spatial and temporal variation in diarrhea occurrence.

The WHO Program for Control of Diarrheal Diseases (CDD) was launched in 1978, to reduce diarrhea-associated morbidity and mortality among infants and young children in lower- and middle-income countries [24]. To address this issue a seven-point action plan promoting immunization, rotavirus vaccination, hygiene, breast-feeding, oral rehydration therapy, and zinc supplementation was established [25, 26]. The national rotavirus immunization coverage in 2022 was 52.3% [27]. Despite these efforts, diarrhea continues to be a serious health issue, accounting for more than 25% of national morbidity across the country and leading to significant geographic disparities [28].

In Ethiopia studies that assess seasonal trends, spatial, temporal, and space-time clusters of diarrhea are not widespread. Many studies have been conducted in different part of Ethiopia including this study area that determines the prevalence of under-5 children’s diarrhea specific to a certain area using logistic regression models but these studies lack the determination of space and time cluster and distribution of under-5 children’s diarrhea at the district level in central Gondar zone. This study includes recent under 5 children diarrhea incidence which is relevant to give a more emphasis on the information that recently exist and provide information for concerning bodies. Also it is methodologically rigorous and provides a comprehensive understanding and it uses SaTScan which has greater power than other available methods for detecting local clusters. Therefore, this study was conducted to fill these gaps and assess the spatial, temporal, and spatiotemporal distributions of diarrhea incidence among children under-5 years of age in the Central Gondar Zone.

## Methods

### Study design and setting

**Fig. 1:**
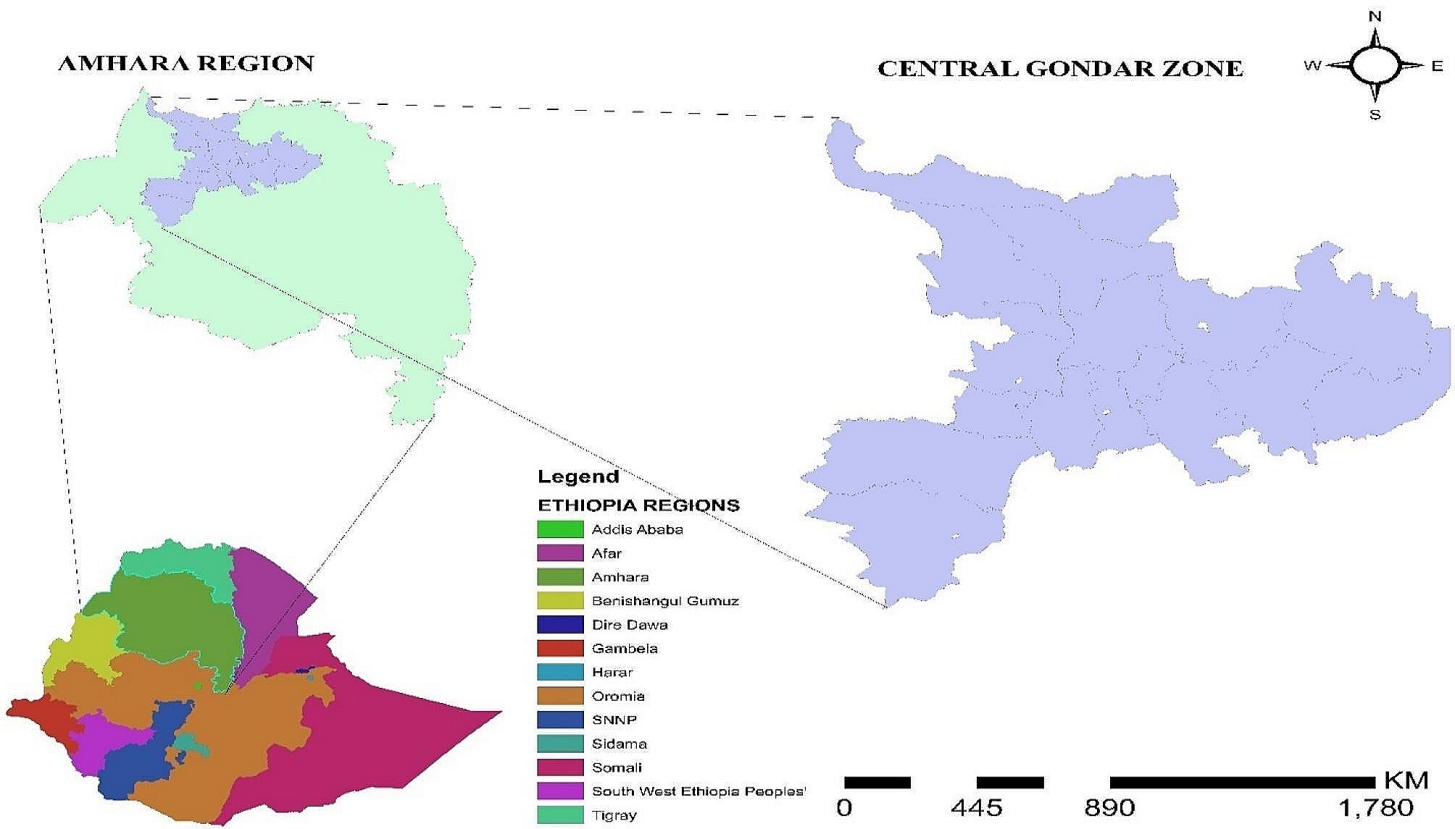
Map of central gondar zone, northwest Ethiopia, Ethiopia. The study area map was created by running on Arc Gis version 10.7.1. (https://www.arcgis.com/index.html)

A time-series cross-sectional study was conducted in the central Gondar zone from 1 January 2019 to 30 December 2022. The Central Gondar Zone is found in the Amhara Region and is geographically located at “170 29’32’’ North latitude and 420 38’25’’ East longitude”. The mean annual rainfall ranges from 875 to 1025 mm and the temperature ranges from 18 to 35 degrees centigrade [29]. It includes 15 Districts and the Gondar city capital of the zone [30]. The boundaries are adjoined with the North Gondar zone in the North, the Awi zone in the West and West Gojam zone in the South, the North Wollo zone in the East, and the South Gondar zone in the Southeast [29]. According to 2014 EC zonal health department data, there is 1 referral hospital, 9 hospitals, 406 health posts, and a total of 796 health extension workers. The total population residing in all districts is estimated to be 2,896,928 [31] and according to the 2014 EC zonal health department, there are 297,241 children under-5 age years of age in all districts (Fig. 1).

### Sample size determination and sampling technique

All confirmed under-5 children diarrheal cases reported from 2019 to 2022 in each district were used. There is no pre-determined sample size to select from the cases reported rather all the cases in each district for each month from 2019 to 2022 was taken and considered as a sample size for the determination of spatial, temporal and spatiotemporal distribution of under 5 diarrhea incidence in Central Gondar Zone.

### Data collection

Data on under-5 children were obtained retrospectively from the Central Gondar Zone Health Department’s diarrhea report. Health facilities treat and record Under-5 children’s diarrhea based on the World Health Organization (WHO) guidelines, which defines diarrhea as passing three or more loose or liquid stools per day, or more frequently than normal, and offering treatment. The data were reported from health facilities to district health offices. District health offices report these cases to the zonal health department. The datasets were subsequently aggregated at the district level and information on the patients was obtained information on cases according to sex category, and time of illness (month and year).

The spatial coordinates (latitudes and longitudes) for each district were obtained from the Ethiopia Statistics Service polygon shape file. Population data for each district for each year were obtained from the Ethiopia Statistics Service.

### Quality control

The data were retrieved from the quarterly surveillance data stored in the Central Gondar Zone Health Department from 1 January 2013 to 30 December 2018. Trained personnel who were familiar with HMIS data management collected the data. The data collectors were informed about the research’s objectives and data collection procedures. All methods were performed in accordance with the relevant guidelines and regulations. After collection, the principal investigator of the study checked the completeness and consistency of the data before analysis. The data were cleaned, edited, checked, and sorted using Excel.

### Data management and analysis

All 15 districts and Gondar city in the Central Gondar zone were included and geo-coded for this study. A shapefile with district boundaries and polygon shapes was obtained from the Ethiopia Statistics Service and each district was georeferenced to its geographic centroid. The centroids provided information on a specific location and enabled us to undertake the district-level analysis. Coordinates were specified using the standard Cartesian coordinate system. The annual incidence of under-5 children in each district and average cumulative annual diarrhea incidence for 2019–2022 were calculated and connected with their respective coordinates. Subsequently, the data were saved in Comma Delimited (CSV).

The ArcGIS 10.8.1 version of the software was used for hotspot detection and construct a map of the study area. In addition, it was used to merge the average cumulative annual diarrhea incidence of under-5 children in each district to the shape file and XY coordinates of the respective districts. The global Moran’s I was calculated for the overall pattern and hot spot analysis was conducted for mapping local clusters. The population data obtained from the Ethiopia Statistics Service were used to calculate the annual diarrhea incidence of under-5 years of age children and used as a known underlying population at risk to fit the Poisson model.

The number of under-5 children’s diarrhea cases in the population at risk was used to calculate the monthly and annual cumulative diarrhea incidence in under-5 years of age children during the specified period. The monthly and annual cumulative incidence of diarrhea among under-5 children of each district and the seasonal trend were calculated using Excel. The data were subsequently plotted to determine the annual fluctuations in the incidence of diarrhea in under-5 children from 1 January 2019 to 30 December 2022.

The discrete Poisson model: was used for this analysis because the data were count data [32]. The number of monthly diarrhea cases, the number of under-5 children population, and the coordinates of the study areas were used as input variables for the discrete Poisson model, with the assumption that the number of cases in each district has a Poisson distribution with a known population of Under-5 children that are at risk for diarrhea. Then, the Poisson data were analysed with purely spatial, temporal, and space-time scan statistics using SaTScan™9.6 software.

Cluster analysis: The scan statistics developed by Kulldorff and SaTScan™ 9.6 software were used to identify the presence of the purely spatial, temporal, and space-time under-5 children’s diarrhea clusters. SaTScan identify a cluster at any location of any size up to a set maximum, which limits the ability of multiple statistical tests, moreover, it also has greater power than other available methods for detecting local clusters [33].

The scan statistics were gradually scanned across time and/or space to identify the number of observed and expected observations inside the window at each location. The scanning window was an interval (in time), a circle (in space), or a cylinder with a circular base (in space-time) for which window sizes were determined, the window with the maximum likelihood was the most likely cluster, and a *p*-value was assigned to this cluster [34].

Spatial scan statistical analysis: A circular window was used to scan the entire study area. The maximum size specifies the percentage of the maximum total population at risk within the scanning window. The maximum cluster size will be set to 50% of the population at risk [19]. The null hypothesis was that disease risk remains the same inside and outside the scanning window in space, while the alternative hypothesis was that the risk within the window is different from that outside the window. A circle with the maximum likelihood ratio and containing more observed cases than expected was identified as the most likely (primary) cluster that was least likely to have occurred by chance [32]. The number of permutations was set to 999 at *P* < 0.05 which was considered to statistical significance [19]. The likelihood function for a specific window is proportional to:

1 $$\left( {\frac{C}{{E[c]}}} \right){\left( {\frac{{C - c}}{{C - E[c]}}} \right)^{C - c}}I(),$$

where C is the total number of cases, c is the observed number of cases within the window and E[c] is the expected number of cases within the window under the null-hypothesis. Note that since the analysis will be based on the total number of cases observed, C − E[c] is the expected number of cases outside the window. I() is an indicator function. The program will be adjusted to scans for clusters with either high or low rates, then I() = 1 for all windows. The expected number of cases in each area under the null hypothesis will be calculated using the formula:

2 $${\rm{E}}\,{\rm{[c]}}\,{\rm{ = }}\,{\rm{p}}\,{\rm{*}}\,{\rm{C/P}}$$

where c is the observed number of cases and p, Under-5 children population in each district while C and P are the total number of cases and population respectively.

Space-time scan statistics: this method was employed to detect clusters in both space and time [19]. It helps to detect clusters that cannot be detected by purely spatial statistics. It is assumed that relative risk of the case was the same within the window compared to outside the window. To detect spatiotemporal clusters a cylindrical window with a circular base was used. The base of the cylinder represents space, as in the purely spatial scan statistic, while the height reflects time [35]. Districts with a significant number of cases within the corresponding time were identified using a *p*-value determined via Monte Carlo simulations. For purely spatial and space-time analyses, secondary clusters in addition to the most likely cluster (primary) were identified using an iterative approach as described in Kulldorff for each purely spatial and space-time scan statistic [18]. The maximum cluster size was set to 50% of the population at risk. A circle with the maximum likelihood ratio and containing more observed cases than expected was identified as the most likely (primary) cluster that was least likely to have occurred by chance [32].

Purely temporal scan statistics: A window that moves in one dimension is used only when the height of the cylindrical window is used as the time dimension. A *p*-value was generated using Monte Carlo simulations. A significance level of *p* < 0.05 was used to identify a significant district. For purely temporal analyses, only the most likely cluster was reported. The scan was used to scan for areas with high rates (clusters).

Spatial autocorrelation analysis: Spatial autocorrelation (Global Moran’s *I*) was used to evaluate whether the disease patterns were dispersed, clustered, or randomly distributed in the study area [36]. It was used to detect the spatial autocorrelation of diarrhea incidence. A calculated Moran’s *I* value close to − 1 indicates disease dispersion, whereas *I* close to + 1 indicates disease clustering and random distribution if the *I* value is zero. A statistically significant Moran’s I (*p* < 0.05) leads to the rejection of the null hypothesis and indicates the presence of spatial autocorrelation [37].

Detection of hotspot areas of Under 5-children diarrhea: Getis-Ord Gi statistics were used to identify cases with either high or low values spatially based on z-scores and *p*-values [38]. Clusters of high values were considered hotspots when the z-score was large and positive, whereas cold spot areas when the z-score value was small and negative [39]. The high/low clustering analysis results were interpreted within the context of a null hypothesis, i.e., “there is no spatial clustering of Under 5 children diarrhea”. When the absolute value of the z-score was large and the *p*-value was very small, the null hypothesis was rejected. The sign of the z-score was considered when the null hypothesis was rejected. A positive z-score value indicated that there was high clustering and a negative z- score value indicated that there was a low clustering in the study area. The *p*-value associated with the 95% confidence interval was 0.05. Statistical analyses were performed and reported using Excel, SaTScan^TM^9.6, and ArcGIS 10.8.1 software.

An Excel spreadsheet was used to describe the data and draw line graphs. Spatial, temporal, and spatiotemporal clusters were analysed using the SaTScan^TM^9.6 program. A map of the significant clusters were generated using ArcGIS 10.8.1 software.

### Ethics consideration

Ethical approval was obtained from the Institutional Review Board of the College of Medicine and Health Sciences, University of Gondar (reference number: IPH/2505/2023). A request letter for the required data was written to the central Gondar Zone Health Department from the Department of Environmental and Occupational Health and Safety and a request letter for the Ethiopian Statistics Service was written from the Institute of Public Health, College of Medicine and Health Sciences, University of Gondar. To ensure the confidentiality of the data, the data were kept secure and were not used for any other purpose.

## Results

### Distribution of counts of under-5 diarrhea patients by type and sex from 2019 to 2022

Monthly diarrheal morbidity data were collected for all 15 study districts and Gondar city from Central Gondar Zone HMIS data. A total of 251,341 under-5 diarrheal cases were reported during the study period. A total of 80.6% (202,587) of under-5 children have functional diarrhea which was the leading cause of under-5 diarrhea. The highest proportion of diarrhea cases was reported among male children with 134,575 cases (53.5%) and between 2019 and 2022 children aged 1–4 years accounted for the largest share with 155,016 cases (61.7%).

### Distribution of incidence of under-5 diarrhea at the district levelfrom 2019–2022

Incidence rates were calculated for all districts every year. This number is a new case divided by the total population and multiplied by 1000. The incidence rate varies from place to place and year to year. Between 2019 and 2022 the highest and lowest incidence rates were observed (249.5 per 1000) in 2021 Gondar city and (82.2 per 1000) was observed in 2018 Tegede district (Table 1).

**Table 1:** Diarrhea incidence among under-five children from 2019–2022 in districts of the central Gondar zone, Northwest Ethiopia

S. *N*	District Name	Yearly diarrhea incidence rate per 1000 under-five children			
		2019	2020	2021	2022
1	Alefa	161.1	173.5	166.0	156.1
2	Chilga 01	139.0	153.9	137.7	125.3
3	Nebaru Chilga	103.5	113.8	115.9	88.7
4	East Dembia	186.9	212.8	190.2	195.2
5	West Dembia	132.6	128.5	122.8	116.3
6	Gondar Zuria	224.4	236.2	246.0	208.7
7	Lay Armacho	176.0	184.0	194.2	167.2
8	West Belesa	162.7	159.9	146.7	140.1
9	East Belesa	134.5	111.3	122.7	120.2
10	Tach Armacho	99.1	110.2	137.3	112.7
11	Central Armacho	90.3	86.6	84.5	87.0
12	Takusa	141.0	135.1	161.7	141.7
13	Tegede	103.6	93.1	105.1	82.2
14	Wogera	163.9	189.7	194.3	176.0
15	Kinfaz Begela	98.0	114.2	109.9	103.0
16	Gondar City	217.6	237.1	249.5	221.7
Average		145.9	152.5	155.3	140.1

### Trends of under-five diarrhea based on annual incidence rate

**Fig. 2:**
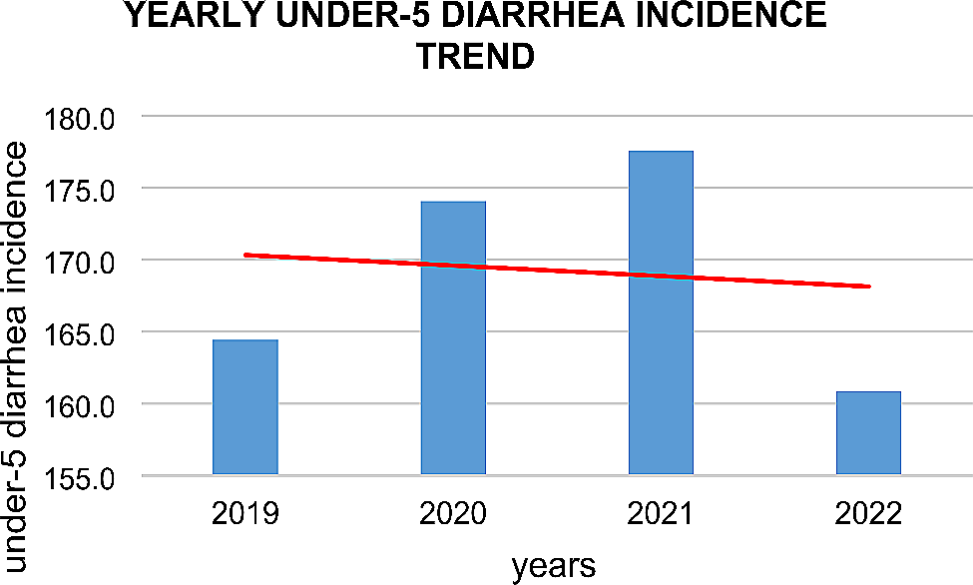
Yearly trend of diarrhea incidence among under-five children from 2019–2022 in the central Gondar zone, Northwest Ethiopia

There was an overall decreasing trend in the incidence of under-5 diarrhea which reached its highest annual incidence in 2021 with 177.6 (66,704) cases, after which it declined and the lowest annual incidence rate was 138.5 (38,079 cases) (Fig. 2).

**Fig. 3:**
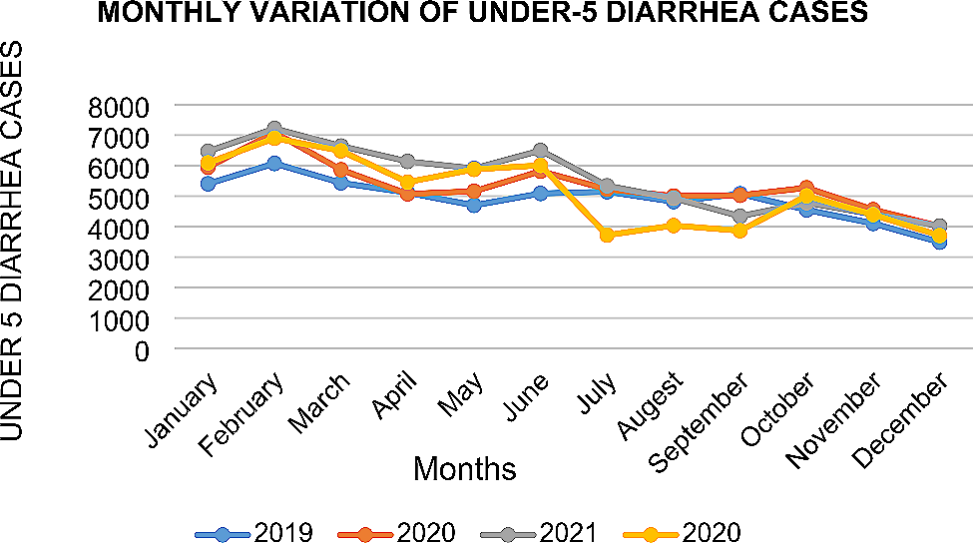
Monthly and yearly variation in diarrhea cases among children under-5 years of age in the Central Gondar Zone, Northwest Ethiopia

There were three under five diarrhea incidence peak months for most years. Overall Fig. 3 illustrates that the incidence of diarrhea among children under-5 years of age in the central Gondar zone decreased from January to December. The peak incidence was recorded in February, followed by June and October (Fig. 3).

### The spatial pattern of the incidence Under-5 Diarrhea

**Fig. 4:**
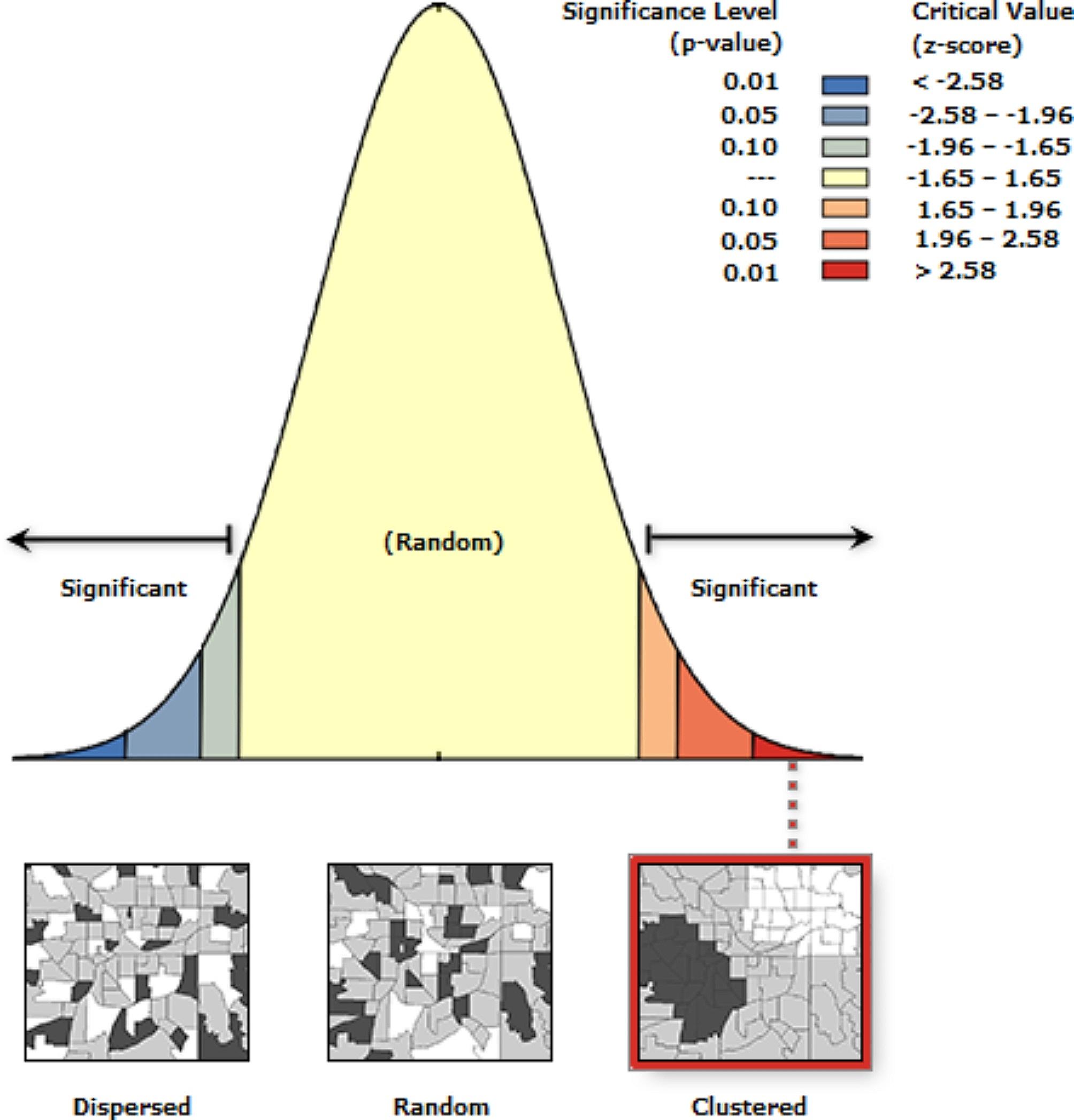
Spatial autocorrelation based on feature locations and attribute values (average cumulative annual diarrhea incidence among under-5children) calculated using the Global Moran’s I statistic across the study area in the Central Gondar Zone, Northwest Ethiopia, January 2019-December 2022

The global autocorrelation results indicated that the incidence of Under-5 diarrhea was clustered (Global Moran’s I = 0.733302 P–value = 0.003375 (Fig. 4).

**Fig. 5:**
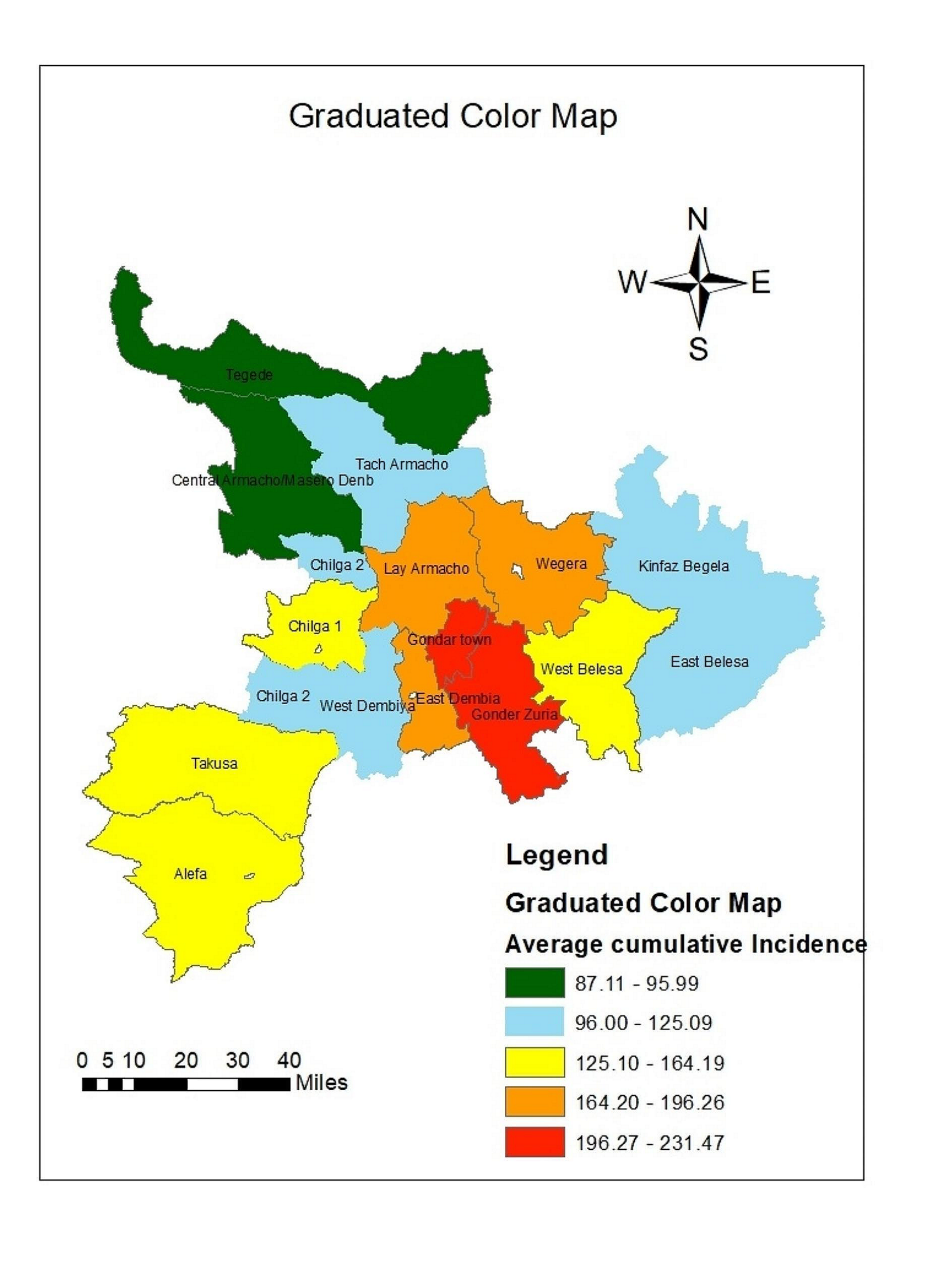
Graduated color (choropleth) map that depicts density based on the average cumulative annual diarrhea incidence among under-5children per 1000 people at risk in the Central Gondar Zone, Northwest Ethiopia, January 2019- December 2022

Districts that had a lower incidence of Under-5 Diarrhea in the Zone are indicated by green color on the map and were clustered around the northern part of the study area, while the highest and second higher Under-5 Diarrhea proportions were indicated by high proportions of dark red and orange color respectively, which are located in the central and southern parts of the study area (Fig. 5).

### Hotspot detection

**Fig. 6:**
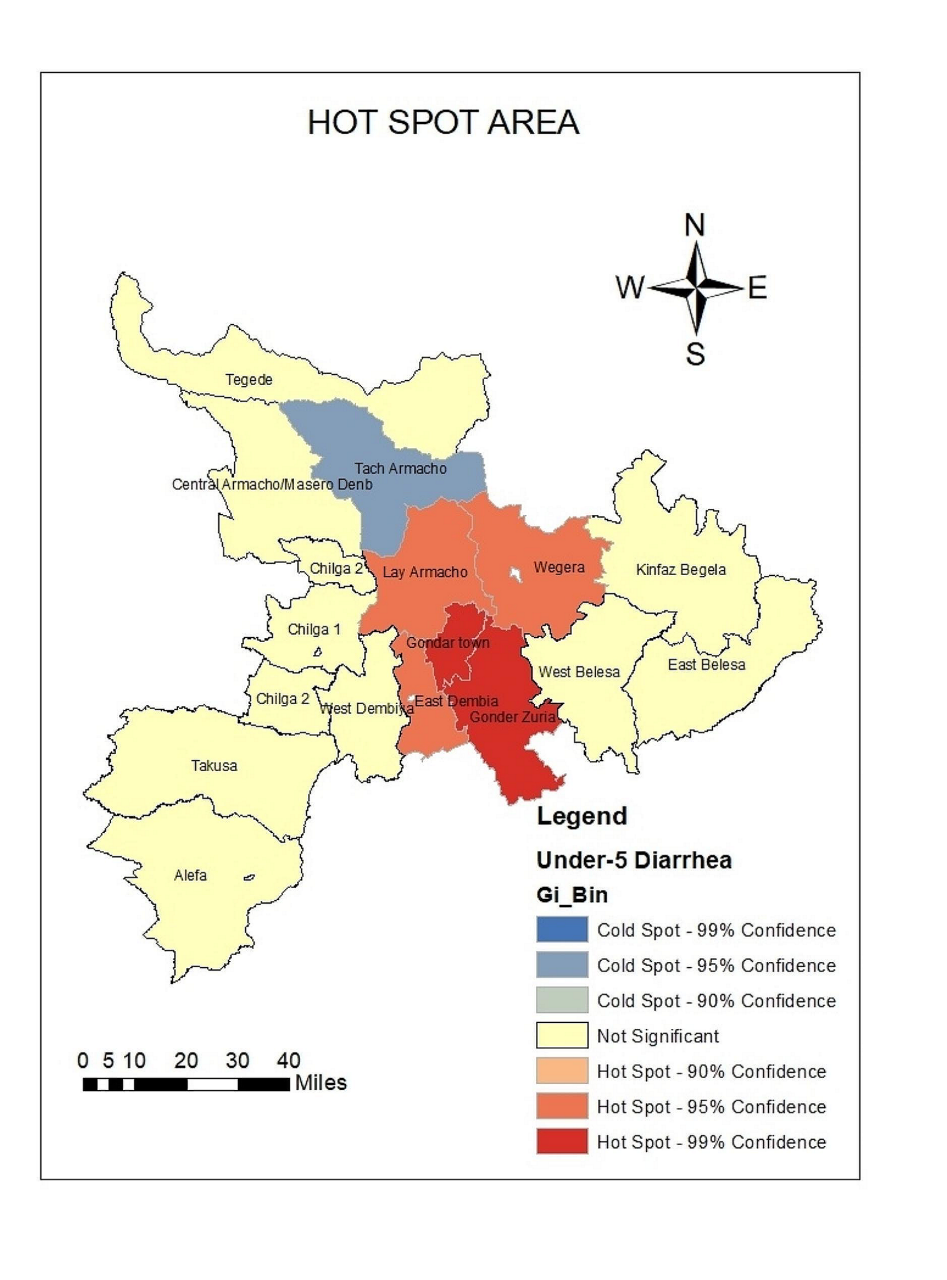
Hotspot detection based on average cumulative annual diarrhea incidence among under-5children in the Central Gondar Zone, Northwest Ethiopia, January 2019-December 2022

Hotspot areas with a high cluster of under-5 diarrhea incidence were identified. A hotspot area with high-rate clusters at 99% confidence was observed in Gondar city and Gondar Zuria. They respectively cover 22.02% (average cumulative annual IR = 231.5) and 12.18% (average cumulative annual IR = 228.8) of the total under-5diarrhea cases reported from January 2019 to December 2022 in the Central Gondar Zone (Fig. 6).

The maximum peak, where spatial clustering was highly pronounced was at a distance of 33750.1845 m with a corresponding Z score of 2.931320 (*p*-value < 0.05). This distance band was used for the analysis of hotspot clusters.

### High-rate spatial clusters

In the study districts, Under-5 diarrhea distribution was found to be clustered. Five high-rate spatial clusters were detected throughout the study period. These clusters were detected hierarchically at Tach Armacho (LLR = 30.53, *p* < 0.0001), Wegera (LLR = 60.78, *p* < 0.0001), East Dembia (LLR = 261.30, *p* < 0.0001), Gondar Zuria (LLR = 1401.65, *p* < 0.0001), and Gondar city (LLR = 3001.02, *p* < 0.0001) (Table 2).

**Table 2:** Significantly high-rate spatial clusters of Under-5 Diarrhea in the Central Gondar Zone, Northwest Ethiopia, January 2019-December 2022

Cluster	District	Population	Coordinates/radius	Obs.*	Exp.*	RR	LLR
1	Gondar city	59,751	12.575528 N, 37.450339 E/ 0 km	55,349	40441.17	1.47	3001.02
2	Gondar Zuria	33,462	12.385233 N, 37.590973 E/ 0 km	30,616	22648.07	1.40	1401.65
3	East Dembia	29,078	12.781798 N, 37.675980 E / 0 km	22,832	19680.98	1.18	261.30
4	Wegera	34,008	12.781798 N, 37.675980 E / 0 km	24,628	23017.36	1.08	60.78
5	Lay Armacho	20,202	12.765897 N 37.369456 E / 0 km	14,571	13673.43	1.07	30.53

*p*-value < 0.0001 for all clusters

RR: Relative Risk;

LLR: Log Likelihood Ratio

Obs.*; Number of observed cases in a cluster

Exp.*: Number of expected cases in a cluster

**Fig. 7:**
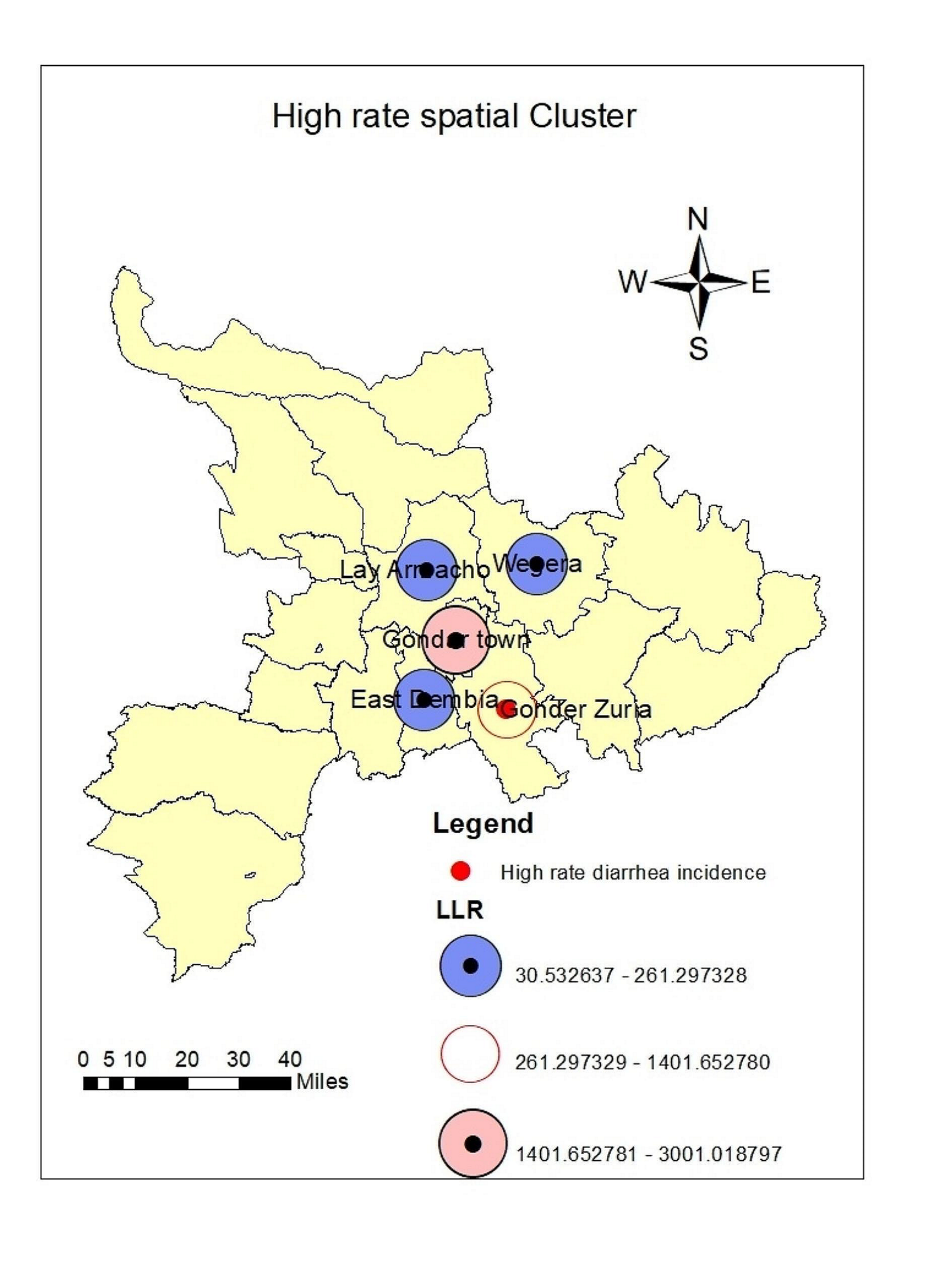
Spatial clustering of Under-5 Diarrhea in the Central Gondar Zone, Northwest Ethiopia, January 2019-December 2022

Based on the value of the likelihood ratio test statistic Color identification of the clusters was ordered according to high-rate clusters (Fig. 7).

### High-rate temporal cluster

Significantly high rates of purely temporal under-5 Diarrhea incidence clusters were observed. These clusters were observed across all districts from January 2019 to December 2022 (LLR = 200.304, *p* < 0.001) (Table 3).

**Table 3:** Significantly high rates of temporal clusters of Under-5 diarrhea incidence in the Central Gondar Zone, Northwest Ethiopia, January 2019 to December 2022

Cluster	District	Time frame	Obs.*	Exp. *	RR	LLR	*p*-value
1	All	January 2020 to December 2021	130,773	125895.91	1.08	189.329	0.001

RR: Relative Risk;

LLR; Log Likelihood Ratio

Obs.*: Number of observed cases in a cluster

Exp.*: Number of expected cases in a cluster

### High-rate spatiotemporal clusters

The spatiotemporal analysis provided further evidence that a greater number of cases than the expected number of under-5 diarrhea cases occurred within a defined place and time. Significant spatiotemporal under-5 diarrhea clusters were detected at Gondar city, Gondar Zuria, East Dembia, Wegera, Lay Armacho, and Alefa between 1 January 2020 and 30 December 2021(Table 4).

**Table 4:** Significantly high-rate spatiotemporal clusters of Under-5 diarrhea incidence in the Central Gondar Zone, Northwest Ethiopia, January 2019 to December 2022

Cluster	District	Time frame	Obs.*	Exp.*	RR	LLR	*p*-value
1	Gondar City	2020/1/1 to 2021/12/30	29,068	20222.94	1.49	1872.856	< 0.001
2	Gondar Zuria	2020/1/1 to 2021/12/30	16,131	11329.96	1.45	946.262	< 0.001
3	East Dembia	2020/1/1 to 2021/12/30	11,746	9867.34	1.20	175.789	< 0.001
4	Wegera	2020/1/1 to 2021/12/30	13,063	11519.57	1.14	104.042	< 0.001
5	Lay Armacho	2020/1/1 to 2021/12/30	7640	6840.05	1.12	46.363	< 0.001
6	Alefa	2020/1/1 to 2021/12/30	5567	5438.77	1.02	1.533	< 0.001

RR: Relative risk;

LLR: Log likelihood ratio

Obs.*: Number of observed cases in a cluster

Exp.*: Number of expected cases in a cluster

## Discussion

A time-series cross-sectional study conducted from 1 January 2019 to 30 December 2022 revealed the occurance of a spatial, temporal, and spatiotemporal distribution of under-five diarrhea in the Central Gondar zone. The incidence of diarrhea in children under-5 years of age exhibited interannual variability across districts and survey years. This finding is supported by a study conducted in southern Ethiopia [40], northwestern Ethiopia [41], resource-limited areas of Ethiopia [19], and Nepal [17] which revealed spatiotemporal variation in under-5 diarrhea. This variation could be due to geographical differences, meteorological factors, the WASH infrastructure, socioeconomic and environmental factors. This study differ from the others since it was done in Central Gondar Zone where there were no such kind of study have been conducted before. This study includes recent under 5 children diarrhea incidence which is relevant to give a more emphasis on the information that recently exist and provide information for concerning bodies. Also it is methodologically rigorous and provides a comprehensive understanding and it uses SaTScan which has greater power than other available methods for detecting local clusters.

The incidence of under-5 diarrhea was greater among males than females in this study with 53.5% (134,575) of male and 46.5% (116,766) of female. These finding are supported by a study conducted on childhood diarrhea in Ethiopia [42] and North West Ethiopia [41] and a study conducted on the health impact of climate change [43] which revealed that males were more at risk for diarrhea than females. The probable reason is that, in Ethiopia, playing outside of their home is allowed for boys, and they begin to participate in economic activities, such as tending to domestic animals in the field when they reach 4 to 5 years of age with their elders [44]. This difference might have contributed to boys having a greater opportunity to wander off into unsanitary surroundings than girls, eventually leading to diarrheal morbidity.

Based on age category children aged less than 1 years accounted for 38% of all cases. This finding is supported by a study conducted in the North Gondar Zone which revealed an that children aged 0–12 months were at risk for diarrhea [45]. This is the time when children start to crawl, walk, and play outside their homes where they come in contact with contaminants from the environment they also start complementary feeding where poor hygiene during food preparation can increase their probability of diarrhea.

In this study area, the total average annual cumulative incidence of Under-5 Diarrhea from 2019 to 2022 was 14.85%. These findings were greater than those of studies conducted in the higher and lower parts of the Amhara region which was 13.5% (95% CI 12.2–14.8%) [46] and in the 2016 EDHS report which was 12% (95% CI 11.39–12.63%) [14]. Dissimilarity might be due to the differences in study designs in which the EDHS used a cross-sectional household survey targeting children who had diarrhea 2 weeks before the survey at the country level. In contrast, this study used a retrospective approach and included children who had diarrhea, visited public health facilities, and received treatment. The differences in the setting, and source of the data, and the variations in the characteristics of the population over the past years contributed to the variations.

The highest -rate of purely spatial clusters between 2019 and 2022 were detected in the Gondar city, Gondar Zuria, Wegera, and East Dembia districts. Population density plays a great role [47] and compared with other districts, Gondar city has the largest population. The displacement of people from surrounding districts to the city increases the crowding and expansion of slum areas in the city. This increases the scarcity of water supply and contact among the population in the city which increases the incidence of diarrhea. Moreover, healthcare utilization for diarrhea might be gteater in cities due to the greater healthcare-seeking behaviors of mothers improved access to healthcare facilities, and an increased number of health facilities which results in high case reporting [48]. In contrast, there might be underreporting of diarrhea cases in rural districts, which contributes to having more cases in this area. These four identified clusters are found closer to one another and one of the important steps of cluster analysis is to detect the aggregation of disease cases and to find evidence of risk factors on which prevention and control activities can be focused. This result provides information for all concerned stake holders and governmental bodies who are interested in the reduction and prevention of morbididty and mortality of under 5 children to provide an intervention and give priorities for the area that are identified as a hot spot areas and increase the health and well being of the community.

This finding showed temporal variation in the overall risk of Under-5 Diarrhea, which indicated that Under-5 diarrhea was not randomly distributed over time. Between 2019 and 2022 a high-rate temporal cluster was found in 2020–2021. This is the year when COVID-19 was an alarming issue in society and everyone’s focus including health professionals, and governmental and nongovernmental bodies who previously worked on diarrhea shifted their focus to COVID-19 prevention and reduction. This contributes to the increase in diarrhea this year. These findings are supported by a study conducted on diarrhea during COVID-19 infection which revealed an increasing number of diarrhea cases ranging from 2 to 50% during the pandemic [49] this is because diarrhea is one sign and symptom of COVID-19. The other reason is that there has been political instability in most parts of the Amhara region and which has created distructions in infrastructure and displaced people, increasing the transmission of diarrhea in this study area.

The spatiotemporal analysis provided further evidence for a greater-than-expected number of under-5 Diarrhea cases arising within a defined place and time. Significant spatiotemporal under-5 diarrhea clusters were detected between 2019 and 2022. There was one primary hotspot and three secondary hotspots of under-5 diarrhea incidence. Primary clusters were detected in Gondar City between 1 January 2021 and 30 December 2022, and the second, third, and fourth statistically significant clusters were identified in Gondar Zuria, Wegera, and East Dembia respectively between 1 January 2020 and 30 December 2021.

## Conclusion

This study revealed spatial, temporal, and spatiotemporal variations in Under-5 Diarrhea incidence in the Central Gondar Zone.A decreasing trend with a seasonal variation in Under-5 Diarrhea incidence which peaks in February, June, and October was observed. To decrease and if possible to illuminate the morbidity and mortality caused by diarrhea it is better if priority intervention is given to the high rate of Under-5 Diarrhea incidence cluster areas identified in this study and if precautionary measures are taken since the incidence of under-5 diarrhea has shown seasonality with three peak points in February, June and October. Further studies to investigate the underlying causes of increased risk in the identified hotspot areas including individual factors, household factors, and environmental factors are recommended to obtain a more inclusive view of Under-5 Diarrhea risk.

### Strength of the study

This study ‘’spatial, temporal, and spatiotemporal variation of diarrhea incidence among Under-5 children” is the first attempt in Central Gondar Zone. Therefore, this study will serve as a baseline for evaluating the progression of Under-5 Diarrhea incidence interventions in upcoming research projects.

This study is methodologically rigorous and provides a comprehensive understanding of the spatial, temporal and spatiotemporal distribution diarrhea incidence among children under-5 years of age. It also serves as a starting point to explore more about what makes hot spot districts hot spot areas and cold spot districts a cold spot area so that we can work on these risk factors.

### Limitations of the study

The data were obtained from a passive surveillance system. This means that all clinical records did not fully capture the level of Under-5 Diarrhea in the districts because some people may not have reported to formal governmental health institutions and instead use traditional therapy or purchase drugs on their own. As a result, the study may not fully reflect the morbidity of diarrhea among children under- five years old in the study area.

This study was supposed to be performed by incorporating host (individual level) factors, household, socioeconomical, other environmental, and organizational factors. Unfortunately, information regarding these variables was not obtained.

## Data Availability

The datasets generated during and/or analysed during the current study are available from the corresponding author upon reasonable request.

## Abbreviations

WHO: World Health Organization
CDD: Control of Diarrheal Diseases
EDHS: Ethiopian Demographic Health Survey
IRR: Incidence Rate Ratio
AIRR: Adjusted Incidence Rate Ratio
LLR: Likelihood Ratio
HMIS: Health Management Information System
CI: Confidence interval
ESS: Ethiopia Statistics Service
SNNP: Southern Nations, Nationalities, and Peoples’.
WASH: Water, Sanitation and Hygiene
